# Buying time: Drug repurposing to treat the host in COVID‐19H

**DOI:** 10.1002/prp2.620

**Published:** 2020-06-23

**Authors:** Jennifer H. Martin, Julian Clark, Richard Head

**Affiliations:** ^1^ Centre for Human Drug Repurposing and Medicines Research University of Newcastle New Lambton NSW Australia; ^2^ Squizzical Pty Ltd Belair SA Australia; ^3^ University of South Australia Adelaide SA Australia

**Keywords:** COVID19, drug repurposing, global collaboration, host response, renin‐angiotensin

## Abstract

In 2016 Fedson stated …. “For almost two decades, leading scientists and health officials have warned that we must prepare for a potentially devastating global pandemic of an infectious disease. Initial concern was focused on …H5N1…. More recently…a devastating outbreak of Ebola virus..(and) several other emerging viruses are believed to seriously threaten global health and global security. To prepare, scientists have been urged to discover new vaccines and treatments for these emerging viruses. At the same time, political leaders have been urged by global health experts to invest millions in a “top down” restructuring of the global health system. This article takes a different view. It focuses on an alternative approach to the scientific discovery of treatments for individual patients, reviews the mechanisms of action and clinical experience with specific drugs that might be useful, and considers whether or not recent lessons regarding this “bottom up” approach to treatment have been learned”.

Now with a new virus and pandemic upon us, Fedson's 2016 comments appear chilling, are cause for reflection on what we have learnt and importantly offer focus on an immediate opportunity in the area of **treating the host** (Fedson DS, Ann Transl Med, 2016;4:421).

The world is facing an aggressive viral attack from nCoV/SARS‐CoV‐2 (COVID‐19 disease). The virus has evolved to use several key proteases including ACE2 to enter cells and tissues. It then reduces ACE2 tissue expression, removing the main pathway to keep the renin angiotensin system (RAS) in check as a modulator of the proinflammatory angiotensin II. Being the pivot to drive end organ disease in COVID‐19, the virus, via angiotensin II and downstream pathways has driven huge morbidity and mortality globally, destroying much of the social and economic fabric of societies. The immediate application of evidence‐based research and its strategic timing (distinct from evidence‐based medicine) is essential to enhance patient survival. For this to occur, a strategy must correctly be aligned with unmet need and this is where an international research strategy can build on success.^1^

Currently there is discussion on two key issues in this COVID pandemic; first, physical isolation, testing (current), and barrier isolation, which is fundamental; second, vaccine and/or antiviral development (possible long‐term future). In physical isolation and impact, some countries appear to be doing very well. Likewise, there is outstanding global research capability and scientists in epidemiology, modeling, immunology, antiviral development, and diagnostics. Within the urgency of a pandemic we are still not achieving treatments with actual efficacy data for prevention, treatment or cure. Realistically, a vaccine may never be developed as planned, or potentially take a further 1‐5 years to complete necessary studies, and finalise the regulatory pharmaceutics dossier, but even then, time is still needed to find funding to manufacture, upscale, and develop supply lines to roll it out globally.^2^ These future hopes are happening against a backdrop of the extremely urgent need to have effective treatments on the wards *here and now*.

We also need an approach to provide a therapeutic insurance with the inevitable easing of social isolation in the absence of vaccines and antivirals. Finally, in seeking effective antivirals one of the barriers to a rational drugs design program for humans is the disconnect between the time and resources required to conduct well‐controlled preclinical and clinical studies and those of biomedical researchers dealing with a global healthcare crisis in real time. The arguments for buying time by utilizing the principles of treating the host are compelling.

In the first instance, we would suggest the world needs to have a much more effective approach to using well‐tested drugs and their dosing schedules that can block a dysregulated innate immune response at clinically tolerated doses. An approach based on treating the host built on sound physiology and pathophysiology, together with thorough administrative data input and accepted principles of drug repurposing based upon pharmacology and clinical pharmacology is needed.

It follows that the immediate unmet need and opportunity sits between these two domains of social isolation and antiviral/vaccine development. It involves pharmacological‐based approaches to treating the *host* (not necessarily the virus) in an acute setting. Treating the host in a viral pandemic context has been highlighted at least 5 years ago.^1^, ^3^

A “***treating the host”*** approach has the potential to enable infected people to survive an acute pulmonary/vascular inflammatory dysregulation with a decreased call on high end health resource. This approach is increasingly recognized as being important to patient care. A critical aspect of COVID‐19 is the constellation of inflammatory processes that become grossly distorted, particularly in the lung, that can be life threatening. The unmet need and opportunity involve the identification of pharmaceuticals to dampen the inflammatory storm in infected individuals with a view to lessen the acute need for intubation and ventilation. It is this need that is displayed vividly in some infected societies across the globe.

The key is therefore not to get overly distracted by the virus, rather the acknowledgment of collateral immediate immune exuberance that is the mortality and health care resourcing problem. And knowing that cytokine storms are quick and overwhelming, we must also focus on therapeutic repurposing for immune modulation. Repurposing itself is a broad discipline; for COVID, drug repurposing to ***treat the host*** is the focus of this Commentary; in distinction, repurposing to identify a novel antiviral effect requires a much longer time frame which will reach fruition outside of this pandemic. Drug repurposing to treat the host includes re‐reviews of previously repurposed drugs in SARS or other treatments for adult respiratory distress syndrome, for example. In this context, the details of repurposing and its limitations in the current era been comprehensively discussed recently.^4^

In order to convert to reality, the opportunity of repurposing drugs to treat the host, we suggest an urgent international drug repurposing clinical research program, governed from a central site, and which in the time of COVID‐19 is based on treating the host. The features of such a program would be:

- An international approach to rapidly identify drugs that treat the host in a pandemic to permit time for vaccine and antiviral development.
- An international approach to coordinating the repurposing of existing drugs in a pandemic based on the principles of treating the host and repurposing existing drugs for new indications.
- Focusing on ***treating the host***, utilizing existing pharmacology and physiology knowledge and observational administrative data, as is in several countries currently but is unlinked to other data and other countries,^5^ and needs to be at scale.
- A fast‐moving program driven by a sense of urgency with the ability to disseminate information to minimize unwanted duplication.
- Designed to incentivize clinicians, scientists, regulators and clinician scientists with ideas, laboratories, infrastructures and clinical trial facilities with skills in repurposing for human use in a pandemic.
- Led by a taskforce of Industry and Research peak bodies, out of a strong pharmacological society.

The focus of this Program would be exclusively on treating the host and not on new chemical entities, antivirals, or vaccines.

Several considerations are evident. Although we usually focus repurposing from the screening aspect and try and translate those into human physiology^6^ there is significant opportunity in identifying repurposing candidates for testing in treating the host from observational data and electronic hospital records of drugs on admission. The value of this approach is maximized when combined with the known pathophysiology of the host's acute inflammatory response to viral infection. An excellent example of this application is apparent in COVID‐19 disease with inhibitors of the RAS. Four observational‐based studies suggest no adverse outcomes and in varying ways a level of advantage with those on RAS inhibitors prior to hospitalisation.^7^, ^8^, ^9^ It is noteworthy that RAS pulmonary dysregulation has been identified by many as an inflammatory hallmark of COVID‐19 disease. Not surprisingly, using clinical trial database searches there are now at least eight clinical trials exploring RAS inhibition in COVID‐19 disease registered on the clinical trials.gov website.^10^

We would suggest that focus in this area be enhanced further to include, for example, global deidentified clinical and administrative data from the SARS‐1 and MERS trials, and observational studies as well as review and adaptation of some of the combined Government, global, and nongovernment organizational funded large‐scale randomised clinical trials (eg, as per those sponsored by World Health Organisation, National Institute of Allergy and Infectious Diseases, Bill and Melinda Gates Foundation). The critical issue would identify repurposing candidates for evaluation in the treating the host context.

Global collaboration from international pharmacology and therapeutic expert bodies such as the British Pharmacological Society, Australasian Society of Clinical and Experimental Pharmacologists and Toxicologists, The American Society for Pharmacology and Experimental Therapeutics, and Japanese Society of Clinical Pharmacology and Therapeutics are also important, and these groups could be a key source of information for Governments in COVID‐19 in a treating the host framework.^11^

In contradistinction, the editors of leading clinical research journals (eg, NEJM, Lancet) have appeared to universally commend investigators who have undertaken and reported on small, sometimes inherently flawed, clinical trials for their initiative and resilience in the face of limited resources and often extremely dire consequences. We would argue that that global program based on treating the host with repurposed therapeutics would provide the essential time needed by those in the vaccine and antiviral development areas to overcome these shortfalls.

Overall then we would thus urge attention be given immediately to coordinated clinical evaluation of existing pharmaceuticals in an international repurposing program targeted toward managing ***the host***, via antagonizing the innate immune pathway or inhibition of the RAS at appropriate dose and timing, in this pandemic.^12^ Such a program can provide adequate time for the development of vaccines, serum‐based approaches or antiviral drugs, and separately will provide a therapeutic insurance with the inevitable easing of social isolation.

## CONFLICT OF INTEREST

There are no conflicts of interest to declare.

## AUTHOR CONTRIBUTIONS

RH conceived of the idea. JM and RH reviewed the literature. JM. RH wrote the article. JC edited and provided advice. Patients or the public WERE NOT involved in the design, or conduct, or reporting, or dissemination plans of our research.
